# Late-Onset Pompe Disease with Normal Creatine Kinase Levels: The Importance of Rheumatological Suspicion

**DOI:** 10.3390/ijms242115924

**Published:** 2023-11-03

**Authors:** Daniela Marotto, Marta Moschetti, Alessia Lo Curto, Anna M. Spezzigu, Miriam Giacomarra, Emanuela M. Marsana, Carmela Zizzo, Giovanni Duro, Paolo Colomba

**Affiliations:** 1 Rheumatology Unit, ASL Gallura, 07026 Olbia, Italy; daniela.marotto@tiscali.it (D.M.); annamaria.spezzigu@aslgallura.it (A.M.S.); 2 Institute for Biomedical Research and Innovation (IRIB), National Research Council (CNR), 90146 Palermo, Italy; marta.moschetti@irib.cnr.it (M.M.); alessia.locurto@irib.cnr.it (A.L.C.); miriam.giacomarra@irib.cnr.it (M.G.); emanuelamaria.marsana@irib.cnr.it (E.M.M.); carmela.zizzo@irib.cnr.it (C.Z.); giovanni.duro@irib.cnr.it (G.D.)

**Keywords:** Late Onset Pompe Disease, misdiagnosis, metabolic myopathy, creatinine kinase value

## Abstract

Pompe disease (PD), also defined as acid maltase deficiency, is a rare autosomal recessive disease that causes glycogen accumulation due to a deficiency of the lysosomal enzyme acid α-glucosidase. An excessive amount of undisposed glycogen causes progressive muscle weakness throughout the body. It particularly affects skeletal muscles and the nervous system, especially in the late-onset phase. Here, we present a clinical case of late-onset PD (LOPD) with normal CK (creatinine kinase) values treated after a misdiagnosis of demyelinating motor polyneuropathy and chronic inflammatory neuropathy. The suspicion of possible fibromyalgia induced the patient to seek a rheumatology consultation, and the investigations performed led to the diagnosis of PD. The patient was investigated for genetic and enzymatic studies. PD was diagnosed using the α-glucosidase assay on DBS. In LOPD, clinical manifestations, such as muscle weakness, exercise intolerance, myalgia, or even high hyperCKemia, often appear as nonspecific and may mimic a wide variety of other muscle disorders, such as limb muscle dystrophies, congenital, metabolic, or inflammatory myopathies. In our case, the patient had CK values in the normal range but with continued complaints typical of PD. An analysis of enzyme activity revealed a pathologic value, and genetic analysis identified the c.-32-13T>G mutation in homozygosis. The association of the pathological enzyme value and mutation in homozygosity with LOPD led to a familial segregation study. Our results contribute to the characterization of PD in Italy and support the importance of rheumatologic attention. This suggests further studies are needed to define the broad clinical and pathological spectrum observed in this disease.

## 1. Introduction

Pompe disease (PD), or glycogen storage disease type II (GSD II), is an autosomal-recessive lysosomal storage disorder caused by acid α-glucosidase deficiency on the GAA gene located on chromosome 17q25 [1,2]. The frequency of PD rates varies by ethnicity and geographic area [3]. Childhood classic (IOPD) is characterized by rapid progression, with an incidence of 1 in 138,000 in Caucasian populations [4]. The incidence of PD is estimated at 1:40,000–100,000 live births in Europe and the United States [5,6,7].

The accumulation of glycogen in various tissues brings broad and typical clinical phenotypes that can be subdivided into childhood classic (IOPD) and non-classic forms (LOPD) [8,9] (Table 1). Classical infantile patients present their first symptoms within the first few months of life with heart failure and profound muscle weakness [9]. Without treatment, most classical infantile patients die within the first year of life [10,11]. In LOPD, initial clinical manifestations, such as muscle weakness, exercise intolerance, myalgia, or even isolated hyperCKemia, often appear unspecific and may mimic a large variety of other muscle disorders such as limb–girdle muscular dystrophies, congenital, metabolic, or inflammatory myopathies [12,13]. Due to these symptoms, the rheumatologist is often the first specialist to be engaged. To date, a dried blood spot (DBS) is used to detect GAA enzyme activity, and if it is reduced, genetic tests are performed to support the diagnosis [14,15].

At present, close to 500 GAA sequence variations have been identified (http://www.pompecenter.nl accessed on 25 September 2023) [8,16], including missense, nonsense, splice-site mutations, and small and large intragenic deletions and insertion [8,17]. The most common is represented by the intronic mutation c.-32-13T>G, which is present in 40–70% of alleles in patients affected with the LOPD form [18].

In this work, we report a clinical study of the Sardegna family, and, in particular, we discuss a LOPD patient. The patient’s medical history reported typical degenerative neuromuscular symptoms treated for years with various nonresponsive therapies. After many hospitalizations and treatments, such as chronic inflammatory neuropathy and depressive anxiety syndrome, he came to the attention of rheumatology with fibromyalgia syndrome. Despite optimal parameters of hyperkemia, we chose, in consideration of symptomatology, to test for Pompe disease.

## 2. Case Presentation

### 2.1. Patients

This study was performed on patients with suspected neuromuscular disorders who attended the rheumatology center of Tempio Pausania Hospital in 2022 in Sardegna, Italy. The biochemical and genetic investigations that led to PD diagnosis were performed at the Centre for Research and Diagnosis of Lysosomal Storage Disorders IRIB-CNR in Palermo and were approved by the Hospital Ethics Committee of the University of Palermo. Signed informed consent was obtained from patients. For the patient, clinical history and laboratory data were collected in a Case Report Form.

### 2.2. Sample Collection

The peripheral blood of the patient was collected using EDTA as an anticoagulant and dried in a specific adsorbent paper spot (DBS). Blood was dropped and allowed to dry for 24 h at temperature room before being sent (with 1–2 days of transport) to a centralized analytical laboratory. Sample storage after use is conducted at a temperature of 4 °C. The same samples from analyzed subjects were used for multiple analyses (enzymatic and genetic assay).

### 2.3. Enzymatic Assay

The analysis of GAA enzyme activity was conducted on DBS, a tool for the diagnosis of PD. The method used was described by Chamoles et al. [19,20] with modifications to improve the precision of the enzymatic activity measurement (unpublished data). The samples were examined using fluorometry techniques. The fluorometric method used substrate 4-methylumbelliferyl-α-D-glucoside [21]. Patients considered positive were those with GAA activity with less than 6 nmol/mL/h.

### 2.4. DNA Extraction

DNA extraction was performed by cutting 3.2 mm diameter punches from the whole DBS spot, corresponding to approximately 3 μL of blood per punch, followed by the addition of 310 microliters of the Digestion Buffer (Buffer G2—Qiagen GmbH, 40724 Hilden, Germany) and 15 microliters of the Proteinase K solution (Qiagen GmbH lot No. 175025821) per each individual sample. DNA extraction was performed using a Qiagen EZ1 advanced XL automatic extractor in combination with the EZ1 DNA investigator kit.

### 2.5. Genetic Analysis-PCR and Sanger Sequencing

Eleven pairs of primers were designed to analyze twenty target regions containing twenty exons of the *GAA* gene. PCR products were purified and analyzed by Sanger sequencing, using an automated DNA sequencer to identify mutations.

### 2.6. Clinical and Laboratory Findings

The index case was a 58-year-old man who presented signs and symptoms such as acroparesthesia, heat and cold intolerance, stiff joints, proteinuria, bone pain, anemia, muscle weakening, recurrent fever, the carpal tunnel syndrome, mucositis, arthritis, and lymphadenopathy. Born preterm at 7 months gestation, the patient was the second-born of an offspring of three children, with all siblings in apparent good health. He was a smoker with the following comorbidities: a history of kidney stones since the age of 16 years with chronic nasopharyngitis, hypertension, myopia, astigmatism, and gastroesophageal reflux.

The patient reported since childhood a history of arthralgias and recurrent gonarthritis for which he underwent evacuative arthrocentesis, abdominal pain with diarrhea but not associated with fever, serositis, and aphthosis. At 27 years, he underwent lithotripsy surgery arising from chronic widespread pain, asthenia, easy fatigability in the upper and lower limbs, particularly after exertion, pain in the lumbar region, muscle cramps at night exacerbated by exposure to cold, frequent falls, and dyspnea.

At 36 years of age, due to the persistence of these symptoms, he was referred to a neurology clinic with the subsequent diagnosis of demyelinating motor polyneuropathy. He was treated with prednisone and gabapentin without benefit.

The same diagnosis, therefore, was questioned at another neurology facility and reformulated after new hospitalization as chronic inflammatory neuropathy and anxious depressive syndrome. The patient was being treated with SSRI (paroxetine) and prednisone without benefit.

Therefore, over the years, he was treated with immunoglobulin and azathioprine with poor efficacy. In 2020, he underwent varicocele surgery.

The patient, at 58 years old, came to a rheumatologist’s observation after being sent by a neurologist with the suspicion of fibromyalgia syndrome.

He reported, in addition to the before-mentioned symptomatology, headaches, the recent onset of paresthesias in the right perioral region, dysesthesias, diplopia, and dysphagia for both solids and liquids.

The patient showed marked and diffuse muscle rigidity and pain, in particular on the mobilization of the left shoulder, rapid fatigue, a mild right wrist tumor, difficulties in walking and climbing stairs, a cautious gait, left claudication, left superficial hypoesthesia conducted with difficulty on the toes and heels, the pronation of the right hand to a Mingazzini neurological test, the hyposthenia of the quadriceps, tibialis anterior and gastrocnemius bilaterally, and more predominately on the left, and osteotendinous reflexes with diffuse hypoevoked and non-evoked Achilles. Moreover, what also made our suspicion of a possible glycogenosis was the patient’s difficulty in switching from the squatted position to the orthostasis with movements that resembled Gower’s sign. In the past, genetic scans for Duchenne muscular dystrophy were included in the diagnostic search plan, but no deletions of the dystrophin gene exons were detected. So, he underwent different clinical investigations.

A hematochemical examination showed the following: blood count (CBC), indices of inflammation, ANA (antinuclear antibody) antiJO-1, antids dna antiSSA, antiSSB, anti-RNP, antiSM, antiLKM1, AMA (anti-mitochondrial antibodies) and ANCA (anti-neutrophil cytoplasmic antibodies) were negative, a normal coagulation profile, creatinine kinase (CK) 90 U/L, LDH, AST 16 U/L ALT 19 U/L, myoglobin 100 ng/mL, Ferritin 50 ng/mL, IgG 706 mg/dL, IgA 108 mg/dL, IgM 95 mg/dL, electrophoresis, a complement test, 24 h of proteinuria, and creatinine clearance, which were all normal. Cholesterol at 220 mg/dL and LDL159 mg/dL were reported.

Electromyography (EMG) showed a picture of modest chronic neurogenic muscle suffering, and nuanced chronic neurogenic muscle suffering was more evident in the distal muscles. In proximal muscles, signs of myopathic muscle suffering were detected. No spontaneous denervation activity was seen.

A cardiac examination reported the diffuse and nonspecific atypia of repolarization. Echocardiogram tests indicated that the left ventricle was not dilated with adequate thickness. No gross changes were detected in segmental kinetics with an ejection fraction (EF) in the normal range. The aorta was not dilated in the explored tracts. The tricuspid aortic valve was detected with mild valvular insufficiency (PHT > 500). E/A was within limits. The right ventricle was undilated and normokinetic (TAPSE:24 mm). Pericardium: nothing relevant. Inferior vena cava evaluation (VCI): not dilated.

Encephalic Magnetic Resonance Imaging and Magnetic Resonance Angiography exhibited radiate crowns, especially on the left, in the posterior arm of the capsule left both internal and in the subcortical white matter at the level of the inferior frontal and cingulate gyri at the level of the knee of the corpus callosum and some gliotic microareolae on a vascular basis were detected. In the subtentorial site, no pathological signal changes were detected. In the supra- and subtentorial site, there were no obvious vascular abnormalities. Median structures were found on the axis. Initial cortico-subcortical atrophy in parietal site at vertex bilaterally but especially right; initial consensual ectasia of the ventricular system, normo-conformed. No obvious aneurysms, malformations, or steno-occlusions to the vessels of the Willis polygon on the left were explorable. Anatomical variants include the focal fenestration of the distal sectors of the basilar artery, the hypoplasia of segment A1 of the right anterior cerebral artery, and the fetal origin of the artery right posterior cerebral artery. Minimal-moderate thickening on the phlogistic basis of the mucosa of the paranasal sinuses with greater involvement of the alveolar recess of the left maxillary was observed. Small retention cysts were found in the mucosa of the vault of the nasopharynx.

**Figure 1:**
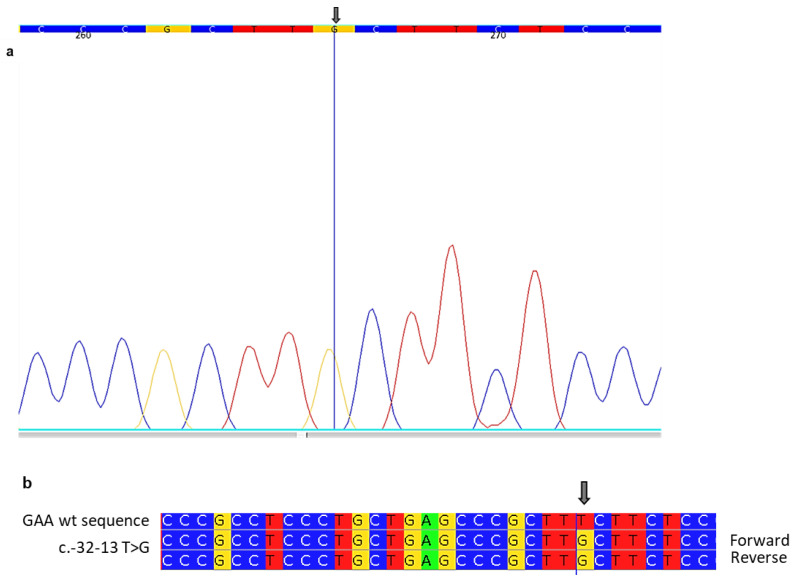
Sanger sequencing shows the mutation c.-32-13T>G. (**a**) In the “Aligne Software” each base corresponds to a color: cytosine is assigned the color blue, guanine yellow, thymine red, and adenine green. A portion of the electropherogram of intron 1 of the GAA gene for the index case in which a mutation is indicated by the arrow. (**b**) A portion of the sequence of intron 1 of the GAA gene (forward and reverse) in a patient aligned with the corresponding sequence of a healthy control (wt). The arrow indicates mutation.

In view of the symptomatology, we decided to test the patient’s entire medical history and various therapeutic treatments without results for PD. A DBS investigation for PD was conducted through enzymatic and genetic studies. The enzyme activity was extremely low, 0.8 nmol/mL/h (normal range > 6 nmol/mL/h). The genetic test of the GAA gene (performed by Sanger sequencing) showed a pathogenic variant c.-32-13T>G in homozygosis localized in the intron 1 region of the GAA gene (Table 2; Figure 1). This variant is known to be associated with the typical LOPD phenotype [22,23].

PD is an autosomal recessive progressive disease, and for this reason, the first diagnostic results obtained led to a family screening. The patient’s father was deceased. The mother, an 81-year-old woman, had normal enzyme activity (6.6 nmol/mL/h) with a c.-32-13T>G mutation in heterozygosis. Our patient was the second born and had two brothers. The analysis of enzyme activity identified pathological values for both subjects. Decreased GAA activity was found at 0.7 nmol/mL/h and 1.6 nmol/mL/h, respectively, for the older and younger brother (Table 3). GAA genetic analysis revealed the presence of a c.-32-13T>G mutation in homozygosis on both brothers. The genetic analysis of the GAA gene was performed using Sanger sequencing.

## 3. Discussion

The proband came to the attention of the Rheumatology Department with a large diagnostic delay of many years, characterized by different diagnoses and different failed therapeutic treatments.

Previously, the formulated neurological observations and the refractoriness to neuropathic treatments associated with corticosteroid and immunosuppressive therapies directed the patient of this study to a rheumatological consultation. This was followed by a series of anamnestic clinical investigations based on the patient’s medical history and symptomatic status, which led to a DBS study for PD investigation.

The enzymatic analysis immediately showed a pathological value of acid α-glucosidase, and the genetics investigation reported the presence of mutations in the GAA gene.

In detail, the mutation detected was c.-32-13T>G in homozygous. The intronic GAA mutation c.-32-13T>G is the most frequent mutation in patients affected by the late-onset form according to 40–70% of the LOPD alleles [24]. The c.-32-13T>G mutation leads to the synthesis of three different aberrant splicing variants. The results of many studies suggest that the effect of this mutation affects the overall splicing efficiency of the pre-mRNA transcript to yield the protein-coding normal isoform [25,26]. Clinical means suggested that the residual of the ‘normal spliced’ mRNA expression could explain the late-onset profile in patients carrying the c.-32-13T>G mutation [18]. This splice-site variant allows the production of low levels of normal GAA and has been traditionally believed to present with a mild, adult-onset phenotype due to the presence of residual enzyme activity. However, there is increasing evidence that the clinical presentation associated with the c.-32–13 T>G variant has a broader spectrum than previously recognized [27,28]. Homozygous c.-32-13T>G patients often have symptoms such as myalgia, exercise-induced muscle fatigue, and, in particular, hyperCKemia [4,29]. The Pompe disease GAA variant database indicates this like most common LOPD pathogenic variants in Caucasians in the splice variant c.-32–13 T>G in intron 1 of the GAA gene, also known as the common IVS1 variant and is very often found in heterozygosity associated with several variants in the GAA second allele, which is necessary to confirm PD diagnosis [22,28].

The first exceptional aspect of our case concerns the levels of CK. Generally, CK concentration is a nonspecific biomarker of muscle disease because there are many pathologic processes characterized by elevated CK levels.

Many laboratory tests are useful when evaluating suspected late-onset PD (such as CK, AST, ALT, LDH, and Glc4). Most late-onset PD patients have elevated CK levels; however, some adults with PD may have CK levels within the normal reference range (22 to 198 U/L) [30]. In this study, the proband had 90 U/L within the norm range values. Therefore, normal CK levels do not exclude the diagnosis of PD, even in patients with mild symptoms. Suspecting PD, even with normal CK, is important because of the availability and remarkable efficacy of therapy.

## 4. Conclusions

Symptoms of these rare diseases, as well as PD, occur in the early years of life, but the delay in diagnosis compromises the possibility of timely treatment. Misdiagnosis is, unfortunately, a factor that prevents the possibility of treatment that can greatly improve the symptoms and course of these diseases. The result of this study on LOPD suggests the importance of early diagnosis in individuals with a high clinical suspicion and symptom presentation attributable to PD, even in those patients with normal CK values. Patients often undergo numerous specialist examinations, therapies of various types, and numerous checks and tests before reaching the correct diagnosis. The diagnostic delay and lack of timely enzyme replacement therapy also result in the clinical worsening of the patient with the subsequent irreversible progression of the disease. In addition, this limits the possibility, once a proband is identified, of being able to study, identify, and then diagnose any other affected family members as well. Extending this genetic study to proband family members is an important element in the early diagnosis and timely administration of therapy in order to decelerate the inauspicious course of this disease. As a rare genetic disease, the diagnostic delay is quite frequent, so it is difficult to be able to identify the specific symptoms and trace them back to the disease. Screening in individuals with suggestive symptoms may provide the best tool for the early identification and diagnosis of PD. An examination of the patient’s relatives has detected other individuals with this rare disease through confirmatory clinical, biochemical, and molecular tests that led to the diagnosis of LOPD.

Thus, the proband study is essential to start the investigation of family members at a later stage and to be able to identify a cluster of affected individuals sharing the same pathogenic and PD allelic variants.

## Figures and Tables

**Table 1:** Clinical presentations of glycogen storage disease type II.

Types of Pompe Disease	Classic Infantile Form	Non-Classic Infantile Form	Adult Onset
Onset	Shortly after birth (usually within the first 3 months)	Delayed onset (within the child’s first year of life)	Any age
Symptoms	Generalized muscle weakness (facial muscles, diaphragm, intercostal muscles) Feeding and hearing disturbances Symptoms of malnutrition ‘Failure to thrive’ Poor weight gain Breathing problems Lung infections Floppiness Head lag Hypertrophic cardiomyopathy that results in heart failure Enlarged liver and tongue	Less severe forms Usually slower progression. Progressive muscle weakness and the delayed development of motor Skills such as rolling over and sitting. Severe respiratory problems due to damage and weakness in the muscles involved in breathing. Abnormally enlarged heart with a lower chance of heart failure compared to the classical form	Milder clinical manifestations and course Respiratory complications due to the weakness of the diaphragm and intercostal muscles Frequent lung infections Most common cause of death is lung failure Myopathy Mobility problems Morning headaches tiredness Weight loss Scoliosis Reduced heart involvement compared to the other form

**Table 2:** Clinvar database information.

Allele ID	Variant Type	Variant Length	Cytogenetic Location	Genomic Location
1013211	single nucleotide variant	1 bp	17q25.3	17: 80104542 (GRCh38)

**Table 3:** Enzymatic and genetic testing for Pompe disease in the index case and familial screening.

Patient No	Sex	Age	Kinship	Mutation	GAA Activity (nmol/mL/h) Normal Range: >6.0
1	M	58	Index Case	c.-32-13T>G homozygous	0.8
2	M	59	Brother of Index Case	c.-32-13T>G homozygous	0.7
3	M	52	Brother of Index Case	c.-32-13T>G homozygous	1.6
4	F	81	Mother of Index Case	c.-32-13T>G heterozygous	6.6

Abbreviations: GAA: acid α-glucosidase.

## Data Availability

No additional data.
